# POCT is a true asset in the emergency department

**DOI:** 10.1186/1757-7241-20-S2-P38

**Published:** 2012-04-16

**Authors:** Niels Jacob Aachmann-Andersen, Poul Jannik Bjerrum, Søren Wistisen Rasmussen, Thomas Andersen Schmidt

**Affiliations:** 1 Emergency Department, Holbæk Hospital, Denmark; 2 Clinical Department, Holbæk Hospital, Denmark

## Background

To determine patient priority and degree of urgency with an objective high-quality evaluation, general Point of Care testing (POCT) was established as a novel facility in the Emergency department at Holbæk Hospital. The hardware consists of AQT-90 Flex (CRP, D-dimer), ABL-800 (Na, K, Hgb, BG etc.) and HemoCue (Leukocytes), thus rendering useful parameters within initial assessment. A clinical biochemist was affiliated and analysis the blood samples taken between 10:00–22:00 hours, i.e. the period where 80% of the total patient flow occurs. The aim of the present study was to investigate whether POCT provides faster blood test results than ordinary clinical laboratory equipment (CLE).

## Methods

On nine randomly chosen days from January 2010 to May 2011 the blood test result time were compared on blood samples extracted between 10:00–22:00 hours. Values are mean±SEM (number of observations). One-way ANOVA was performed to assess overall significance and followed by a Tukey studentized range test.

## Results

The response time (minutes) from blood samples where extracted until the samples were analyzed were for CLE 73±2(166), AQT-90 Flex 30±0(136), HemoCue 17±1(117) and ABL-800 11±0(197) and did not vary significantly during the observational period. The response time for CLE analyses was longer than for POCT throughout the period by as much as 49 minutes on average (p<0.001). However, there was no significant difference between the response time for CLE and POCT when evaluating the time from the arrival of patients until the test results were obtained, even though this response time decreased by 40 and 52%, respectively during the observational period.

## Conclusion

Overall, POCT seems to have led to a higher capacity for patient-turnover, with more patients discharged directly from the emergency department, fewer re-hospitalisations, improved efficiency, and faster results for important lab tests. However, when comparing response time from arrival of the patient to when the test result is established, POCT is a not significant contributor. This indicates that in order to benefit from POCT the time before taking blood samples should be reduced to a minimum meaning that overcrowding needs to be controlled.

